# Exploring the relationship between perceived social support and college students’ autonomous fitness behavior: Chain mediating effect test

**DOI:** 10.3389/fpsyg.2022.1036383

**Published:** 2023-02-01

**Authors:** Na Li, Shuqiang Zhao, Chanjuan Liu, Kun Dai, Wenjian Huang

**Affiliations:** School of Physical Education, Shandong University of Science and Technology, Qingdao, China

**Keywords:** perceived social support, autonomous fitness behavior, mental toughness, exercise self-efficacy, college students

## Abstract

**Objective:**

This study aims to explore the effect of perceived social support on college students’ autonomous fitness behavior, and the mediating role of mental toughness and exercise self-efficacy.

**Methodology:**

A survey participated by 985 college Students (average age, 19.55) was conducted by applying the following scales: The Perceived Social Support Scale, the Adolescent Self-Government Behavior Scale, the Mental Toughness Scale, and the Exercise Self-efficacy Scale (ESES).

**Results:**

(1) Perceived social support can directly and positively predict autonomous fitness behavior, mental toughness, and exercise self-efficacy; mental toughness can directly and positively predict exercise self-efficacy. Likewise, perceived social support, mental toughness, and exercise self-efficacy can positively predict autonomous fitness behavior. (2) The indirect effect of the path with mental toughness as the mediating variable is 0.078, the indirect effect of the path with exercise self-efficacy as the mediating variable is 0.122, and the indirect effect of the path with mental toughness and exercise self-efficacy as the mediating variable is 0.082. (3) The total of all indirect effects is 0.282, and the effects of the three indirect pathways account for 18.25, 28.62, and 19.37% of the total, respectively.

**Conclusion:**

The perceived social support can indirectly predict college students’ autonomous fitness behavior through the independent mediating effect of mental toughness and self-efficacy, as well as the chain mediating effect of the two. The claim that mental toughness and exercise self-efficacy perform a chain-mediate role in the positive effect brought by perceived social support on autonomous fitness behavior has been supported. This study revealed the relationship and mechanism between perceived social support and college students’ autonomous fitness behavior and further improved the research on the impact of perceived social support on college students’ autonomous fitness behavior.

## Introduction

Facilitating student’s autonomous fitness behavior is one important facet of a society’s strategy to promote health and wellbeing. To accomplish the goal of “Big Health” in the new era, “*the Outline of the Healthy China 2030 Plan*” proposes to “maintain and guarantee people’s health throughout the whole life cycle.” Under the development framework of “health first,” making prevention beforehand has become the most scientific and economical strategy for dealing with the “window period” of sub-health. The physical fitness of college students has become not only the key to building sports power but also the demonstration of Comprehensive National Power (CNP), which is crucial to the future of the country. In this regard, ensuring a healthy and resilient youth reserve force has become a common and incumbent duty of society (Jie, 2015). Autonomous fitness behavior is restricted by internal and external factors, among which the encouragement and support of important others, such as parents, relatives, and friends, are the key factors (Atkins et al., 2015). Perceived social support affects individuals’ cognitions and concepts. It is also an important psychological resource for college students when faced with difficulties and setbacks, which will affect the generation of healthy sports behavior as an inexhaustible motivation. Therefore, exploring the relationship between perceived social support and autonomous fitness behavior is an important approach to improving the sports participation of the youth and solving their physical problems. In recent years, research on social support and physical fitness behavior has received extensive attention from scholars. Tao and Solmon (2010) highlights that adolescents supported by their families can participate in sports more actively, and their physical activity behavior can be improved through positive social interaction (Salvy et al., 2007, 2008). Existing studies have focused on addressing issues of social support related to student health behavior (Chen et al., 2018; Wang et al., 2019; Lu and Shilan, 2020). However, the relationship between perceived social support and autonomous fitness behavior and research within the underlying mechanism is rarely touched upon. Based on this, this study aims to explore the relationship between college students ’ perceived social support and autonomous fitness behavior, and variables that may mediate this relationship, to provide theoretical and practical support for intervention.

### The relationship between perceived social support and autonomous fitness behavior

Autonomous fitness behavior refers to what the behavior subject consciously chooses, optimizes, and compensates for behavioral objectives and means. As a physical fitness activity with a certain intensity, fitness behavior is carried out for health, leisure, socializing, etc. (Rui et al., 2012), which demonstrates the self-esteem and self-regulation abilities of the behavior subjects based on self-determination theory. Self-determination theory holds that the internal power of autonomous choice behavior can be mutually transformed with the degree of autonomy of social support, that is, the internal and external motivation can be mutually transformed (Edward and Richard, 1985). As the strongest autonomy, autonomous motivation determines the initiative and motivation of human behavior (Ryan and Connell, 1989), reflecting the basic characteristics of high-quality self-determinism and the closest to internal (Deci and Ryan, 2000; Johnmarshall et al., 2002).

Among them, the positive attitude generated by understanding the support from important people and the environment is the internal driving force of college students’ autonomous fitness behavior. Yimin et al. (2001) believe that there is a high correlation between psychological factors such as youth’s attitude toward sports and their behavior toward sports. Namely, social support is both objective and subjective support from all parties in society, reflecting the degree and quality of the relationship between the individuals and society (Wei et al., 2016). Perceived social support is the college students’ expectations and feelings of support from significant others. After feeling and understanding the source of social support, the resulting positive, stable, and lasting power accordingly promote college students’ emotional involvement in sports participation (Wang et al., 2019; Beihe et al., 2022). Among them, family support has been proven to be a positive predictor of youth physical activities (Draper et al., 2015). On the one hand, understanding that supportive behavior from parents and friends promotes college students’ participation in regular physical activities and helps to maintain an active lifestyle (Tao et al., 2012). On the other hand, when coaches support, the athletes will more actively adhere to long-term exercises (Pelletier et al., 2001). While the students perceive the support of physical education teachers, their level of participation in out-of-school sports activities can be positively predicted (Hagger et al., 2013). In addition, support from classmates and peers is also positively associated with their sports participation (Bronikowski et al., 2016), and the lack of friends or parental support is considered a barrier to college students’ sports participation (Abdelghaffar et al., 2019). It can be seen that the individual subjective perceived impact is greater than the actual support obtained (Ting, 2015). The model of social support in promoting students’ exercise behavior reveals that the basic psychological need, autonomous motivation, and exercise intention have a chain mediation effect in their exercise behavior promotion (Beihe et al., 2022). On this basis, this study tries to explore the operation mechanism of the resulting mental toughness and self-efficacy in its influence, so as to provide a reference for the formation of college students’ autonomous fitness behavior and the promotion of lifelong physical exercise behavior. Therefore, Hypothesis 1 is put forward: perceived social support can positively predict college students’ autonomous fitness behavior.

### The mediating role of mental toughness

Mental toughness is an ability to adapt well to unfavorable situations, such as stress, adversity, and threats, as a hotspot in positive psychology research. Scholars have analyzed the content and elements of mental toughness by building process models and hierarchical models. Olsson et al. (2003) believes that mental toughness includes factors such as personal abilities and personality traits, family, and social support systems. As individuals with abilities and characteristics to cope with adversity in life, college students with higher mental toughness are supposed to have higher cognition and better emotional control level in the face of setbacks. At the same time, students feel and understand the support and protection from family and society, will form protective psychological potential resources, also make them able to successfully deal with and change adverse circumstances, with more positive perseverance and belief in response, and have better social adaptability. Sports behavior is closely related to mental health, and understanding the positive psychological experience stimulated by social support is also better performed in sports. College students with a strong social support network can actively respond to and control the situation in sports, better maintain a high level and excellent sports performance, and adjust themselves to reduce anxiety at the psychological advantage, which strengthens the psychological resilience of young people. Through more effective regulation of emotional information, higher mental toughness affects the devotion to physical exercise and presents a higher competence to deal with emergencies in competitions (Jiejin and Chengyue, 2021). The positive benefits of mental toughness can cope with pressure and difficulties in the process of fitness, and it ensures the formation of stable emotions and adaptability, and can better regulate stressful behaviors and more active participation in physical fitness activities, and then promote the formation and maintenance of independent fitness behavior (Hao and Qianyu, 2022). In this regard, Hypothesis 2 is proposed: perceived social support affects college students’ autonomous fitness behavior by improving mental toughness.

### The mediating role of exercise self-efficacy

As the core of the social cognitive theory, self-efficacy refers to an individual’s perception and belief about whether to complete the expected behavioral goals and has high predictive power for maintaining and changing individual behaviors (Bandura, 1999; Benyu and Feiyue, 2008). Self-efficacy is the determinant of behavioral motivation and the core of behavioral activities, it can regulate individuals’ cognition, attitude, emotion, etc., and influence their behavioral attitudes by providing motivation (Judge and Bono, 2001). Self-efficacy, as a key variable predicting individual behavior and the most closely related mediator of behavioral changes (Yongzhan et al., 2012), has a stable and consistent relationship with sports behavior (Duda et al., 1998). It is also closely related to regular physical activity (Bauman et al., 2012), and is an important factor in predicting physical fitness behavior (Min, 2012), college students with high exercise self-efficacy are more likely to participate in physical activities regularly (Hagger et al., 2001). In addition, support from family, friends, and peers can significantly and positively predict college students’ autonomous fitness behavior (Pingan, 2020), and it indirectly affects the level of physical fitness by improving self-efficacy (Ran et al., 2022). Self-efficacy is the mediating effect between social support and sports behavior (Hui, 2019). Another study has also proved that friend support, self-efficacy, and physical fitness behavior are related (Shangjian, 2016), and social support is positively correlated with self-efficacy and youth physical fitness behavior (Liang and Congyi, 2022). Based on this, Hypothesis 3 is proposed: perceived social support affects college students’ autonomous fitness behavior by improving exercise self-efficacy.

### The chain mediating effect of mental toughness and exercise self-efficacy

Studies have shown that mental toughness is positively correlated with self-efficacy (Chen et al., 2020; Penghua et al., 2022; Qi et al., 2022), and mental toughness positively predict self-efficacy. This correlation is not only manifested in the fields of management and pathology, studies have found that active physical exercises can help college students obtain higher psychological toughness, which in turn improves their self-efficacy (Chaohui, 2020; Lei et al., 2021; Wu et al., 2022). Since there is a significant positive correlation between perceived social support and mental toughness and self-efficacy, perceived social support not only has a direct impact on self-efficacy but also indirectly affects self-efficacy through mental toughness as an intermediary (Kun et al., 2022). At the same time, self-efficacy has a direct promotion effect on college students’ physical fitness behavior, and higher self-efficacy affects physical fitness participation by generating positive emotions and strong will (Jun and Han, 2018). The rationale that the chain-mediated model can be explained by self-determination theory, based on the self-determination theory, as an effective factor to predict and change college students’ exercise behavior, social support creates conditions for the formation and transformation of college students’ motivation to exercise independently (Sheldon and Krieger, 2007; Malik et al., 2015; Beihe et al., 2022). When college students perceive that their basic needs are met, this satisfaction will affect their independent motivation, exercise intention, and exercise behavior in turn, and form healthy behavioral beliefs, attitudes, and participation intentions, including the improvement of mental toughness and exercise and exercise self-efficacy, therefore, perceived social support becomes an effective predictor of autonomous fitness behavior. Accordingly, Hypothesis 4 is proposed: mental toughness and exercise self-efficacy play a chain mediating role between perceived social support and autonomous fitness behavior.

**FIGURE 1:**
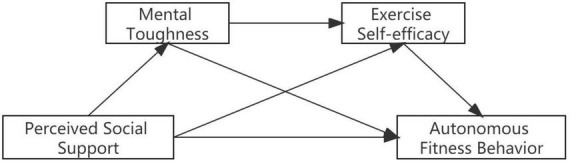
Conceptual model.

Based on this, this study intends to construct a chain mediation model (Figure 1), aiming to explore the influence of perceived social support on college students’ autonomous fitness behavior, as well as uncover the mediating role of mental toughness and exercise self-efficacy, to provide a theoretical reference for the formation of college students’ autonomous fitness behavior and lifelong physical exercise.

## Materials and methods

### Procedure and participants

According to the administrative region of China, five provinces were selected from the Northern (Shandong, Liaoning), Central (Anhui, Henan), and Southern (Jiangsu, Zhejiang, and Guangdong). From each province, three classes of a university are randomly selected. Questionnaires were distributed through online surveys, and 1,018 college students were investigated. After excluding invalid questionnaires (such as regular responses and data omitted), 985 valid questionnaires were finally obtained. Among them, there were 298 boys (30.3%) and 687 girls (69.7%), with an average age of 19.55; 237 (24.1%) were students in provincial and prefecture-level cities, 355 (36%) in county-level cities, and 393 (39.9%) in townships and villages.

This survey was approved by the school leadership, tutors, and participants, and all of the participants were college students, following the principle of being voluntary, confidential, and anonymous. Taking a collective test method, the questionnaire with control variables such as age, gender, students, and school location of the participants was collected from 3 May 2022 to 1 July 2022. Each questionnaire took approximately 5–10 min to fill in and all the invitees participated voluntarily, and the study was undertaken following an appropriate ethical review (Supplementary Table 1).

### Measures and instruments

#### Perceived social support scale

The perceived social support was assessed by the Multidimensional Social Support Scale (MSPSS) developed by Blumenthal et al. (1987) which was translated and revised by Qianjin (2001) It is a tool for measuring the degree of personal understanding of social support from various aspects, which contains three dimensions (family support, friend support, and other support) and 12 items (e.g., when I have problems, some people will appear around me, for example, teachers, classmates, and relatives), and adopts a seven-level scoring method, with 1 being completely inconsistent and 7 being completely consistent. The higher the average score is, the higher the level of support from others in society subjectively felt by individuals (Qin and Luyao, 2022). According to the applicability study of Chinese students, the results showed that the internal consistency of the scale was good. Its internal consistency coefficient is α = 0.93. Spearman–Brown half-reliability coefficient was 0.905, and the internal consistency reliability of family support, friend support, and other support in the subscales were 0.818, 0.875, and 0.846. The internal consistency coefficient of the scale in this study was α = 0.972.

#### Autonomous fitness behavior scale

Autonomous Fitness Behavior Scale was compiled by Chinese scholar Fang Rui et al. (2012), using the “*Self-determination Scale for autonomous Fitness Behavior*,” which included 3 dimensions such as self-determination (e.g., I had fun with my friends during my exercise, α = 0.859), self-supporting (e.g., I often watch sports games live or on television, α = 0.786), and self-regulation strategies (e.g., I have stuck to my fitness program for a long time, without any supervision, α = 0.876), 43 items, using a five-level scoring method, 1 being very inconsistent, 5 being very consistent. The Spearman-Brown half-reliability coefficient was 0.802, 0.743, and 0.789, and the Guttman fold half reliability coefficient was 0.802, 0.740, and 0.789, all reaching the acceptable standard. It has been tested as an evaluation tool with high reliability and validity and can be used to evaluate the autonomous fitness behavior of Chinese students. In this study, the internal consistency coefficient of the questionnaire was α = 0.983.

#### Mental toughness scale

The mental toughness scale was compiled by Chinese scholar Yueqin and Yiqun (2008), based on the conceptual model of mental toughness and went through the localization test. It was used with a total of 27 items (e.g., Failure has always discouraged me), using a Likert five-level evaluation, with 1 for complete non-conformance, 5 for complete agreement, to assess 5 dimensions ranging from goal focus, interpersonal assistance, family support, emotional control to positive cognition. The internal consistency reliability of the scale, a preliminary test was α = 0.85, a retest was α = 0.83 and in this study, the internal consistency coefficient of the scale was α = 0.938, also the test used the Resilience Scale (RS) developed by Wagnild and Young (1993) as a calibration marker, showing a moderate correlation α = 0.53.

#### The exercise self-efficacy scale

The exercise self-efficacy scale (ESES) was developed by Kroll et al. (2007), and revised by Yanjin et al. (2016). The scale contains 10 items (e.g., if I try my best, I can always solve the problems I encounter during the exercise), the Likert four-level score is adopted, with 1 being completely inconsistent, and 4 being completely consistent. Through the Sinicization of the English sports ESES, the internal consistency coefficient of the Chinese ESES scale is α = 0.879, split half reliability coefficient is *r* = 0.858, and the test–retest reliability is 0.657. The internal consistency coefficient of the scale in this study is α = 0.948.

#### Research procedure and statistical analysis

(1) IBM SPSS26.0 statistical software was used for data analysis, including (1) descriptive statistics and correlation analysis of perceived social support, autonomous fitness behavior, psychological resilience, exercise self-efficacy, and other variables. (2) The coefficient of internal consistency and the internal consistency reliability were measured using Chronbach’s alpha values method. The statistical step is Analysis-Scale-Reliability Analysis.
(2) To inspect the possible common method deviation, Harman single factor tests are more often used, after the statistical “analysis-dimensionality reduction-factor analysis,” and all items were extracted and included in a single factor unrotated exploratory factor analysis. There were 10 factors with eigenvalues greater than 1.0, and the explained variance of the largest factor was 39.31%, which is less than the 40% criterion recommended by Hair et al. (1998). Therefore, there is no obvious common method bias in this study.
(3) Model 6 in the macro program PROCESS of SPSS was used to conduct the mediating effect test (Hayes, 2013). Major test: the direct effect relationship between perceived social support and autonomous fitness behavior; mediating effect of mental toughness and exercise self-efficacy; the chain mediating effect of perceived social support and autonomous fitness behavior.
(4) AMOS26.0 was used to test the fitting degree of the mediating model between perceived social support and autonomous fitness behavior.

## Results

### Descriptive statistical and correlation analysis

Table 1 shows the average value, standard deviation, and correlation coefficient of perceived social support, autonomous fitness behavior, mental toughness, and exercise self-efficacy, indicating that the correlation between the variables has reached a significant level. Among them, perceived social support and autonomous fitness behavior, mental toughness, and exercise self-efficacy were significantly and positively correlated (*p* < 0.01).

**TABLE 1:** Descriptive statistics and correlation analysis.

	Sex	Age	Perceived social support	Autonomous fitness behavior	Mental toughness	Exercise self-efficacy
Sex	1					
Age	0.093	1				
Perceived social support	-0.038	−0.033	1			
Autonomous fitness behavior	0.185**	−0.032	0.647**	1		
Mental toughness	0.143**	−0.057	0.596**	0.657**	1	
Exercise self-efficacy	0.196**	−0.019	0.542**	0.826**	0.572**	1

*N* = 985. **p < 0.01.

The correlation degree of perceived social support, autonomous fitness behavior, mental toughness, and exercise self-efficacy of adolescents of different genders is shown in Table 2. Perceived social support of female is significantly higher than that of male. But autonomous fitness behavior of male is significantly higher than that of female, similarly, mental toughness and exercise self-efficancy are also higher in men than in women.

**TABLE 2:** Differences in gender.

	Gender	Number	*M* ± *SD*	*t*
Perceived social support	Female	687	5.50 ± 0.95	1.37***
	Male	298	5.42 ± 1.26	
Autonomous fitness behavior	Female	687	3.45 ± 0.65	-6.72***
	Male	298	3.74 ± 0.89	
Mental toughness	Female	687	3.45 ± 047	-5.49***
	Male	298	3.64 ± 0.77	
Exercise self-efficacy	Female	687	2.82 ± 0.54	-7.15***
	Male	298	3.06 ± 0.68	

*N* = 985. ***p < 0.001.

### Significance test of mediation effect

In this study, model 6 of the SPSS plug-in PROCESS compiled by Hayes (2013) was used to test the mediation effect. Table 3 shows that perceived social support can directly predict autonomous fitness behavior (β = 0.43, *p* < 0.001), and hypothesis 1 is established. Perceived social support positively predicted mental toughness (β = 0.29, *p* < 0.001). Also perceived social support positively predicts exercise self-efficacy (β = 0.16, *p* < 0.001), and mental toughness can directly and positively predict exercise self-efficacy (β = 0.38, *p* < 0.001). Perceived social support, mental toughness, and exercise self-efficacy could positively predict autonomous fitness behavior at the same time (β = 0.14, *p* < 0.001; β = 0.26, *p* < 0.001; β = 0.75, *p* < 0.001).

**TABLE 3:** Regression analysis of the relationship between variables.

Effect	Item	Effect	SE	*t*	LLCI	ULCI
Direct effect	Perceived social support -autonomous fitness behavior	0.14	0.015	9.57***	0.113	0.171
Indirect effect	Perceived social support-mental toughness	0.29	0.014	21.38***	0.267	0.321
	Perceived social support-exercise self-efficacy	0.16	0.018	9.19***	0.128	0.197
	Mental toughness-exercise self-efficacy	0.38	0.033	11.15***	0.311	0.444
	Mental toughness-autonomous fitness behavior	0.26	0.029	9.151***	0.208	0.322
	Exercise self-efficacy -autonomous fitness behavior	0.75	0.026	28.88***	0.694	0.795
Total effect	Perceived social support -autonomous fitness behavior	0.43	0.018	23.97***	0.390	0.458

*N* = 985. ***p < 0.001.

**FIGURE 2:**
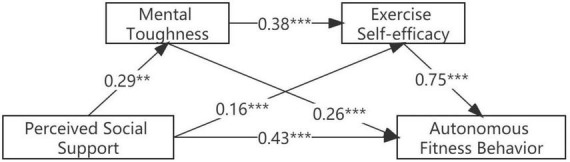
The chain-mediated mediation path of perceived social support to college students’ autonomous fitness behavior. **p < 0.01, ***p < 0.001.

Further chain mediation model test results are shown in Table 4 and Figure 2. The indirect effect of the path with mental toughness as the mediating variable is 0.078 (95% CI = [0.054, 0.102]), the indirect effect of the path with exercise self-efficacy as the mediating variable was 0.122 (95% CI = [0.090, 0.159]), and the indirect effect of the path with mental toughness and exercise self-efficacy as the mediating variable was 0.082 (95% CI = [0.061, 0.105]), the total of all indirect effects is 0.282 (95% CI = [0.245, 0.319]), and the effects of the three indirect paths account for 18.25, 28.62, and 19.37% of the total, respectively. Therefore, the chain mediating effect of perceived social support on autonomous fitness behavior is established.

**TABLE 4:** Bootstrap analysis of significance test of intermediary effect.

Influence path	Indirect effect		95% confidence interval		The ratio of total effect
		**Boot SE**	**Boot LLCI**	**Boot ULCI**	
Total indirect effect	0.282	0.019	0.245	0.319	66.24
Ind1	0.078	0.012	0.054	0.102	18.25
Ind2	0.122	0.018	0.090	0.159	28.62
Ind3	0.082	0.011	0.061	0.105	19.37
Ind1–Ind2:	-0.044	0.023	-0.096	-0.000	–
Ind1–Ind3:	-0.005	0.015	-0.037	0.025	–
Ind2–Ind3:	0.039	0.024	-0.008	0.087	–

Ind1: Perceived social support→mental toughness→autonomous fitness behavior; Ind2: Perceived social support→exercise self-efficacy→autonomous fitness behavior; Ind3: Perceived social support→mental toughness→exercise self-efficacy→autonomous fitness behavior.

### Structural equation modeling analysis

In this study, the structural equation model test is performed by using the maximum likelihood estimation method to test the fit degree between the measurement model and the actual data collected. The results show that χ^2^/df = 10.286, RMESA = 0.097, CFI = 0.908, NNFI = 0.914, GFI = 0.919, and AGFI = 0.921. All the fitting indexes are higher than the standard of 0.9. It shows that the mediating model of perceived social support and autonomous fitness behavior fit quite well (Table 5).

**TABLE 5:** Model fit index of the mediating role of perceived social support and autonomous fitness behavior.

χ^2^/df	CFI	AGFI	GFI	NNFI	RMESA
10.286	0.908	0.921	0.919	0.914	0.097

## Discussion

### The relationship between perceived social support and autonomous fitness

This study shows that perceived social support can significantly and positively predict college students’ autonomous fitness behavior, which verifies Hypothesis 1 and is consistent with the research of other scholars (Malik et al., 2015; Aiqun et al., 2020; Lixia et al., 2020; Pingan, 2020). Perceived social support is social support that is perceived by college students to meet basic fitness needs, which means that the help and support provided are accepted and understood by college students, affect their basic psychological needs of fitness, promote positive investment and form a stable, positive, enduring awareness of sports participation. As social psychology believes, social support can guide individuals to continuously adjust and improve beliefs, thereby enhancing behavioral ability by promoting efficacy, therefore, perceived social support on college students’ physical fitness behavior is self-promoting, which is consistent with the results of scholars in the field of training (Xiuying et al., 2022). Perceived social support can bring out a benign evaluation of unknown events, and then effectively deal with possible negative emotions in the process of behavior. That is, social support is protective and can enable individuals to provide effective encouragement through a positive coping attitude and improve their self-confidence and cognitive evaluation, which is based on the stress buffer theory to understand the positive efficacy of understanding social support (Kawachi and Berkman, 2001). The obtained positive feedback in sports not only improves one’s own adaptability but also reduces the negative experience in the behavior process (Lin and Yeh, 2014), enabling students to participate in the physical activity process more happily.

The social support perceived by college students has formed a positive attitude, which in turn affects behavior and belief. This psychological mechanism can refer to the Planned Behavior Theory (Lerman and Kern, 1983) and Hedonic theory (Diane, 1984; Courty and Engineer, 2019). The social support perceived by college students generates their positive emotional attitudes, and changes in emotional cognition affected their behavioral beliefs. Because of the susceptibility of college students, their emotional attitudes are easily influenced by the mainstream culture of society which brings out a sense of belonging (Lerman and Kern, 1983), which becomes the key variable guiding their motivation and then dominating their behavior outcomes (Weiner, 1985), and supporting orientate the development of behavioral intention. At the same time, based on the cognitive judgment of the features and significance of physical activities (Magee and Tiedens, 2006), the behavioral attitude of physical fitness is generated, and then the positive self-fitness behavior.

### The mediating role of mental toughness

This study shows that mental toughness mediates the relationship between perceived social support and college students’ autonomous fitness behavior. Perceived social support is an important factor affecting mental toughness. A higher level perception of social support produces a more positive emotional experience, maintains a stable emotional and psychological state, which in turn affects mental toughness and promotes healthy behaviors, this result is also supported by related research (Vansteenkiste et al., 2005; Yu et al., 2005; Xinhui et al., 2022). A positive attitude is an important characteristic of high mental toughness (Yongtao et al., 2015). Mental toughness is also a necessary psychological factor for athletes to achieve excellent performance (Gucciardi and Jones, 2012). The greater the perceived social support from coaches and other significant others, the richer the positive experience and the higher the self-exercise behavior are exhibited. In a variety of sports contexts, they can maintain better self-confidence and lower anxiety, the belief in dealing with adversity, and stronger personal strength and support (Yueqin and Yiqun, 2008). The positive emotions generated by high mental toughness can adjust and adapt to unfavorable environments, such as pressure and setbacks encountered by individuals and actively adjust their psychological emotions (Lu and Shilan, 2020). It showed higher emotional strength and tenacity in the process of exercise, and also adjusted themselves to participate in sports more actively (Dapeng et al., 2013; To et al., 2015). At the same time, regular sports activities, in turn, are the “pressure buffer” to improve mental toughness. Therefore, we should cultivate college students’ mental toughness through sports activities and then improve their autonomous fitness behavior.

Mental toughness development theory (Richardson, 2002) regarded that the changes in mental toughness were the developing process of the psychological balance from damage to reshape. Stressors for individuals triggered unadaptable cognition, which broke the original psychological balance, and the individuals were forced to transfer the protective factors, establishing a new equilibrium state by reinventing the psychological cognitive style and faith. As a protective factor of stressor response (Ziluo and Zhiyu, 2022), mental toughness is innate, the dynamic model of mental toughness also points out that when adverse factors appear in life, protective factors reduced or moderated their effects in time. The protective factors include the following (1): various external protective resources provided by society and family, can provide support through safety, emotion, and belonging, cultivate college students’ capabilities, affect their physical and mental health, and help them to adapt better. (2) As protective factors of resilience, internal resources, such as self-esteem and emotion regulation, have a positive effect on resilience (Mestre et al., 2017; Xueqin, 2021; Yongb et al., 2021). Internal and external protective factors complement each other in their growing process. When external support is perceived, internal protective factors such as self-esteem and emotion are enhanced, and they will be less vulnerable to risks, showing better psychological adaptability.

Through the mechanism analysis, social support was found as an important source of protective factors of mental toughness, and the key to improving it is to improve the level of internal and external protective factors (Han, 2015), including the social support perceived by college students (Guoliang, 2013), their mental toughness can be enhanced by increasing social support for their physical fitness behaviors (Herbert et al., 2018). Mental toughness plays an intermediary role in the autonomous fitness behavior of college students. Students with strong mental toughness have more positive emotional experiences and more confidence when engaging in physical fitness activities, which further enhances the autonomous fitness behavior of college students.

### The mediating role of exercise self-efficacy

This study found that exercise self-efficacy plays a mediating role between perceived social support and college students’ autonomous fitness behavior, which also verifies Hypothesis 3 and is supported by existing research results (Hui, 2019; Liang and Congyi, 2022). In the field of social cognitive psychology, self-efficacy, as a core element, has the highest predictive power, maintaining and changing individual behaviors by monitoring, evaluating, and regulating emotions (Bandura, 2001). As an individual’s psychological motivation, when the individual perceives his own coping ability, as an incentive mechanism, the increased self-efficacy can improve the sense of self-control and self-evaluation (Bandura, 1977), and become an effective factor in predicting the autonomous fitness behavior of college students, this result is consistent with studies abroad (Malina et al., 2016; Lafuente et al., 2019). Self-efficacy regulates and stimulates individual behaviors based on existing beliefs through the synergy of cognition, belief, and emotion (Bandura, 2003). At the same time, self-efficacy, as an important internal trait and cognitive factor of one’s own initiative, make full use of external information sources to learn the necessary skills and to have the confidence and ability to achieve specific achievements. Therefore, individuals with high self-efficacy can still persevere in finding solutions in unfavorable environments, while low self-efficacy prompts them to escape from the predicament as soon as possible. Relevant studies have shown that among college students, social support can affect their sports behavior and self-efficacy (Biao et al., 2022; Wang et al., 2022). Yakun et al. (2020) also confirmed that self-efficacy enhances self-consciousness, can positively predict sports behavior and can improve the level of sports participation promoted by improving self-efficacy.

In sports participation, college students perceive the benefits of exercise behavior and change in health information, and their behavioral beliefs can affect their behavioral attitude and willingness to participate. Willingness, as the basis and premise of behavior, has a direct predictive effect on behavioral outcomes (Carpenter, 2008; Cantell et al., 2014). So exercise self-efficacy as a beneficial variable to adjust the mental health of the youth, can not only increase the adherence and persistence of the physical exercise behavior but also improve the positive emotional experience. Based on this, positive exercise motives should be built, and detailed plans should be made to enhance college students’ exercise self-efficacy and their willingness to participate.

### The chain mediating effect of mental toughness and exercise self-efficacy

The study further found that perceived social support can indirectly predict college students’ autonomous fitness behavior through the chain mediating effect of mental toughness and self-efficacy. The dual engines that activate college students’ autonomous fitness behavior are internal psychological appeals and externally perceived support, and internal and external interactions promote college students to have healthy consciousness and behavior (Baolin et al., 2018). The more support they perceive from important people in physical fitness activities, the stronger their self-esteem protection is. The self-confidence they build makes it easier to eliminate the negative emotions in the process of physical activities, thus improving their mental toughness and promoting their self-awareness and autonomy in exercise. This is consistent with previous research (Xiao et al., 2014). When one’s self-confidence is established, and that fitness behavior is confirmed to have a positive effect, this health belief produces a high sense of self-efficacy and then realizes the occurrence or change of the expected behavior (Bandura, 1999). This result is also applicable to the field of management (Xiling et al., 2018; Lafuente et al., 2019), further illustrating the positive relationship between mental toughness and exercise self-efficacy. Self-efficacy is a protective factor of mental toughness, which can significantly and positively predict mental toughness (Yunbo, 2018; Jing et al., 2022), college students with higher mental toughness have higher self-efficacy (Juzhe et al., 2011), and those with higher self-efficacy can form correct perception and understanding of themselves, thus improving mental toughness (Linlin, 2020). Individuals with high mental toughness have a higher sense of self-efficacy. When faced with negative emotions, they can exert their positive self-confidence, fearlessness of difficulties, and high self-repair ability, thereby indirectly promoting the improvement of autonomous behavior (Penghua et al., 2022).

Therefore, perceived social support can promote college students’ autonomous fitness behavior, their mental toughness and exercise self-efficacy play an intermediary role in promoting college students’ autonomous fitness behavior, and the mediation model constructed in this study is feasible, all of which to some extent reveal the internal mechanism of perceived social support to improve college students’ autonomous fitness behavior, also brings certain guiding value for improving the practice of college students’ autonomous fitness behavior. To improve autonomous fitness behavior, the willingness of participating in sports should be continuously stimulated, the construction of the joint system of home school, and society be explored, by building an integrated cooperative communication mechanism; and the cultivation of psychological resilience be strengthened by constructing the grid psychological education pattern. Also from the subconscious perspective of values, college students should stimulate their motivation to participate, improve their self-efficacy, and make full preparations for the health of college students in the future. At the same time, physical educators should coordinate various factors in all aspects, and give full play to the premise of social support efficiency, encourage students to participate in sports activities, because the law of physical activity is the most important factor to maintain a healthy life state.

## Limitations and future prospects

This study explores the relationship between perceived social support and college students’ autonomous fitness behavior. By constructing a chain mediation model, it reveals the internal mechanism of perceived social support on college Students’ autonomous fitness behavior which has both important theoretical and practical values for understanding college Students’ autonomous fitness behavior. It also provides a premise for further research on how to improve autonomous fitness behavior and inspires the prevention and intervention of college Students’ physical health. However, this study needs to be improved further: First, the results of this study are only limited to the mediating role of mental toughness and exercise self-efficacy in perceived social support and college students’ autonomous fitness behavior. There are other mediating variables such as family environment, self-control, and parenting style, which need to be further explored in the follow-up research. Second, the objects of the study are college students over 19 years old, and the sample source is relatively single. It is necessary to be cautious when the variables such as perceived social support and autonomous fitness behavior are extended to other age groups. In order to obtain higher misrepresentations, future research needs to expand the sample range; Third, this study adopts the self-report method of college students to conduct the survey, and the possible common method bias will affect the research results, future research should obtain data from teachers, peers, parents, and other channels to further explain the relationship between variables. Finally, perceived social support has a positive predictive effect on college students’ autonomous fitness behavior, and mental toughness and exercise self-efficacy play a part of the intermediary role. The research results suggest that the improvement of college students’ health is inseparable from the belief support given by parents, friends, and educators. Schools, families, and communities should provide sufficient sports resources to meet the psychological needs of young people, improve self-confidence and self-awareness of fitness, the positive self-perception evaluation formed during fitness is the key to improving self-exercise fitness behavior among disadvantaged and negative adolescents. In the process of promoting college students’ autonomous fitness behavior, perceived social support should focus on psychological needs and provide various types of support that can be perceived, which can not only improve college students’ psychological enthusiasm and satisfaction but also improve self-efficacy, which are two significant paths to improving autonomous fitness behavior.

## Conclusion

Perceived social support can independently predict college students’ autonomous fitness behaviors. Mental toughness and exercise self-efficacy play a significant mediating role between perceived social support and autonomous fitness behaviors. There are three mediating paths, namely, the separate mediating effect of mental toughness, the separate mediating effect of exercise self-efficacy, and the chain mediating effect of mental toughness and exercise self-efficacy. Therefore, families, schools, society, etc., should provide social support that can be understood, felt, and accepted by students. Physical education teachers should take positive encouraging measures to improve students ‘ideological character of hard work and courage, which can not only improve their psychological resilience, but also improve students’ exercise self-efficacy, and then improve college students’ independent fitness behavior.

## Data availability statement

The original contributions presented in this study are included in the article/Supplementary material, further inquiries can be directed to the corresponding authors.

## Ethics statement

The studies involving human participants were reviewed and approved by the School of Physical Education at Shandong University of Science and Technology of China, and all participants signed an informed consent form and were paid for their participation. The participants provided their informed consent in written form to participate in this study.

## Author contributions

NL and SZ designed the study, collected and analyzed the data, and wrote the manuscript. CL and WH did the translation and revision. KD and NL investigated and revised the manuscript supervision and funding acquisition. All authors contributed to the article and approved the submitted version.
